# An Overview of Potential Alternatives for the Multiple Uses of Per- and Polyfluoroalkyl Substances

**DOI:** 10.1021/acs.est.4c09088

**Published:** 2025-01-24

**Authors:** Romain Figuière, Luc T. Miaz, Eleni Savvidou, Ian T. Cousins

**Affiliations:** † Department of Environmental Science, Stockholm University, Stockholm SE-10691, Sweden

**Keywords:** functional substitution, regrettable substitution, alternatives assessment, PFAS-free, database

## Abstract

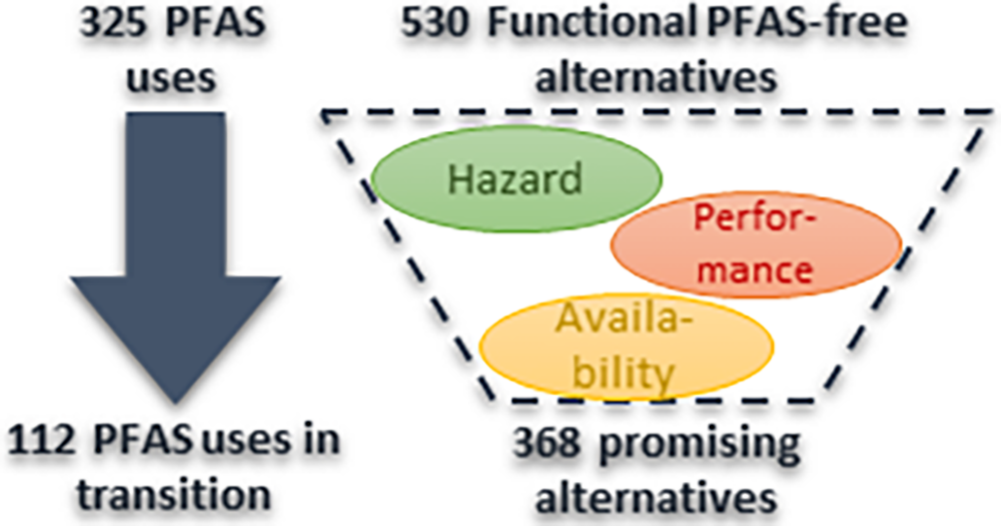

Per- and polyfluoroalkyl substances (PFAS) are used in a wide range of different industrial and consumer applications. However, due to their extreme environmental persistence and their impacts on human and ecosystem health, PFAS have been subject to many regulatory activities, including initiatives to incentivize industry to transition toward PFAS-free alternatives. Although efforts have been made to map all uses of PFAS, work is still needed to provide an overview of their potential alternatives. Based on the functional substitution approach, this study develops an online database that documents all known uses of PFAS, describes the functions provided by PFAS in these uses, lists potential alternatives that can deliver equivalent or similar functions to PFAS, and evaluates the suitability of the identified alternatives to replace PFAS. Overall, the database lists 325 different applications of PFAS across 18 use categories. In total, 530 PFAS-free alternatives are identified. Based on a screening of potential concerns of the identified alternatives, their performance compared to PFAS, and their availability on the market, it is concluded that potentially suitable alternatives to PFAS are available for 40 different applications. For 83 applications, no alternatives could be identified at the time of the study and should be the focus of further research activities.

## Abstract 2

This study provides an overview of the availability of suitable alternatives to PFAS across their multiple uses.

## 1 Introduction

Per- and polyfluoroalkyl substances (PFAS) make up a group of synthetic fluorinated organic substances used for many decades in a wide range of industrial and consumer applications. Their widespread use is due to their special properties including thermal and chemical stability as well as the omniphobic (both hydrophobic and oleophobic) nature of perfluoroalkyl chains.^1,2^ The group of PFAS comprises a high number of substances with a huge diversity of physicochemical properties^3,4^ and (eco)toxicological potential.^5,6^ Recently, four member states of the European Union (Denmark, Germany, The Netherlands, and Sweden), and Norway submitted a restriction proposal under the Registration, Evaluation, Authorisation, and Restriction of Chemicals (REACH) Regulation (EC No 1907/2006) in order to restrict the manufacture, placing on the market, and use of PFAS.^7^ This broad grouping approach is based on the extreme persistence of PFAS,^8^ combined with other hazardous properties that trigger additional concerns. The restriction proposal aligns with the general objective of the European Commission to “ensure [···] that the uses of PFAS are phased out in the EU, unless they are proven essential for society” as stated in the Chemical Strategy for Sustainability (CSS).^9^ This is in line with the conclusion of a previous study which suggested that the essential-use concept could be implemented to identify uses of PFAS, which are not essential for society in order to speed up their phase-out.^10,11^ However, as the essential-use concept was not implemented at the time of the restriction proposal, it was not used in the preparation of the dossier.^12^ In parallel to these regulatory efforts on PFAS in the EU, several states of the United States have also proposed to restrict the use of PFAS.^13−16^ Additionally, perfluorooctanoic acid (PFOA) and perfluorohexanesulfonic acid (PFHxS), and their related compounds are listed in Annex A of the Stockholm Convention, which obliges the Parties of the treaty to take measures to ban the production and uses of those substances.^17^ Parties must also take measures to restrict the use of perfluorooctanesulfonic acid (PFOS), its salts, and perfluorooctane sulfonyl fluoride (POSF) as they are listed in Annex B of the Stockholm Convention.^17^ Although these various regulatory activities have many differences in terms of scope and their application, they all have the desirable outcome of encouraging companies to transition toward PFAS-free alternatives.

Although the substitution of a toxic chemical is the most effective means to reduce its associated risk,^18^ replacing it with an alternative can result in unforeseen consequences and regrettable substitution, as demonstrated in recent examples. For instance, when perfluorooctanoic acid (PFOA) was phased out by manufacturers, it was for example substituted with hexafluoropropylene oxide dimer acid (HFPO–DA) for its use in the manufacture of fluoropolymers.^19^ However, recent studies have demonstrated that concentrations of HFPO–DA in drinking water are now increasing and that HFPO-DA can cause human health effects.^20−22^

Alternative assessment frameworks have been developed to avoid such regrettable substitutions.^23^ They can be defined as processes for “identifying, comparing, and selecting alternatives to chemicals of concern (including those in materials, process, and technologies) on the basis of their hazards, performance, and economic viability”.^24^ The functional substitution approach has been developed in order to efficiently screen and compare a broader range of alternatives.^25^ The developers of the functional substitution approach encourage the assessor to define the chemical functions, end-use functions, and functions as a service of a chemical in a specific use to be able to identify a broader range of substitutes capable of providing comparable functions. By doing so, they argue that the alternatives assessment will go beyond drop-in substitutes, providing more substitution options and thus helping to avoid regrettable substitution.^25^ Furthermore, this approach can provide improved knowledge of the specific purposes of the chemicals of concern in specific uses, which can help regulators to target functions for which safer alternatives are truly needed.^25,26^

Although efforts have been made to map the uses of PFAS,^2,27^ work is still needed to gather information on potential PFAS-free alternatives to determine if those uses of PFAS can be phased out.^28^ As highlighted by Ateia and Scheringer (2024), an open data sharing platform on uses of PFAS, the functions they deliver, and their potential alternatives is needed to maximize the collective knowledge on PFAS-free alternatives, and to accelerate the transition away from PFAS.^28^ Therefore, this study was developed as part of the European project ZeroPM in order to (1) identify the applications where PFAS are used; (2) describe the functions provided by PFAS in each application in order to understand the purpose they serve by following the functional substitution approach; (3) identify potential alternatives that could deliver similar functions; and (4) evaluate the suitability of the alternatives to determine whether PFAS can be substituted. The goal of this study is not to debate whether all PFAS represent the same risks for human health or the environment nor to determine if every use of PFAS should be phased out. The main purpose is rather to provide an overview and preliminary analysis of the potential PFAS-free alternatives that are already available as well as to highlight where they are not available yet.

## 2 Methods

### 2.1 Definitions

#### 2.1.1 Definition of PFAS

Following the definition of PFAS used by the Organisation for Economic Co-operation and Development (OECD)^29^ and by the general REACH restriction proposal^7^ without their exclusion criteria, any substance containing a fully fluorinated methyl (−CF_3_) or methylene (−CF_2_−) group in its molecular structure was considered as PFAS for the purpose of this study.

#### 2.1.2 Definition of Uses

**Figure 1:**
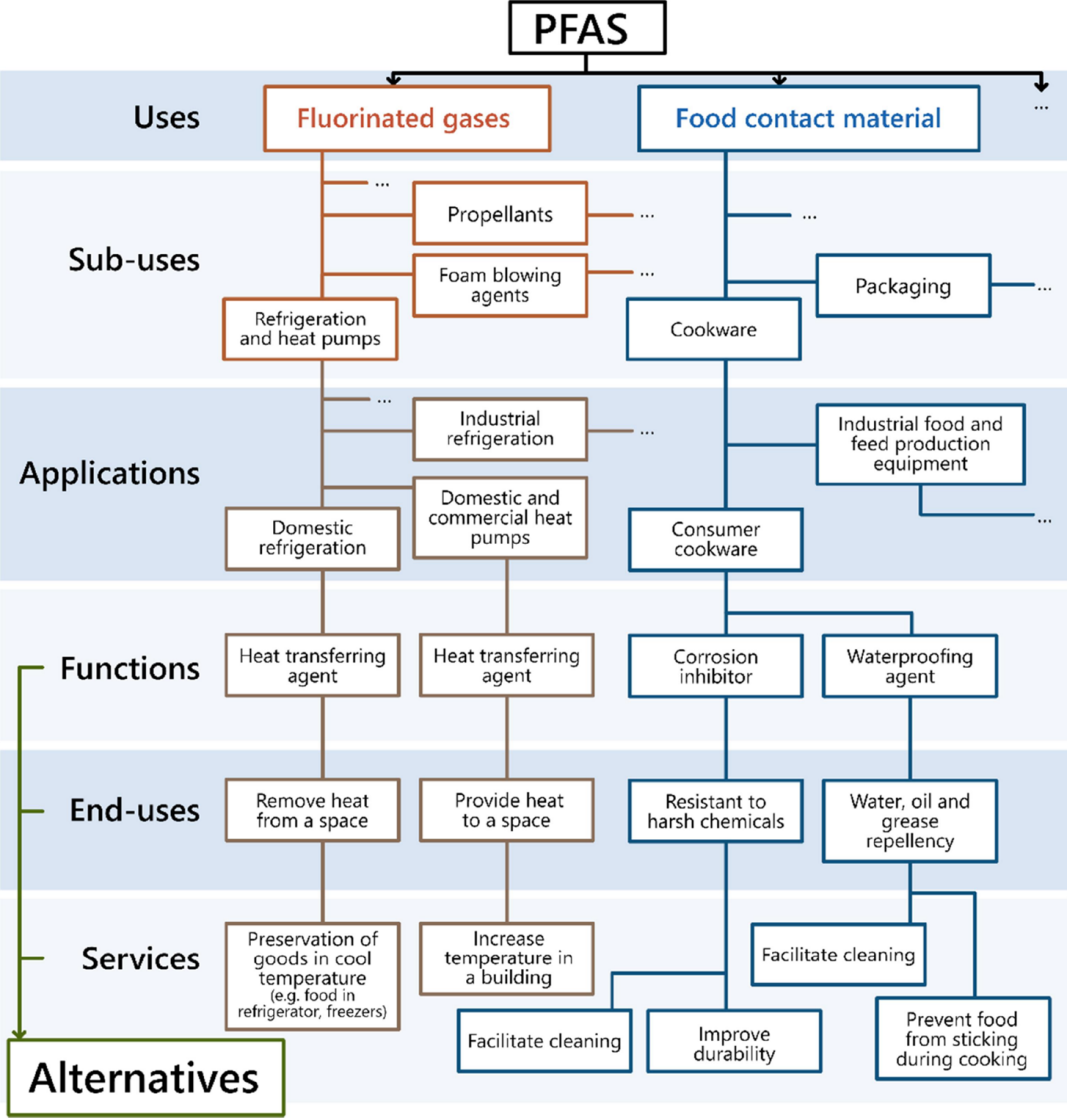
General structure of the database. Note: this figure illustrates the structure of the database by using specific examples of PFAS used as fluorinated gases and in food contact materials. As it is not possible to represent all functions for all applications in all sub-uses and use categories of PFAS, “···” was used to indicate that more uses, sub-uses, applications, and functions are covered in the database than those listed in the figure.

As illustrated in Figure 1, PFAS were sorted into different use categories, sub-uses, and applications. In this study, the term “use category” refers to the sector or the type of products PFAS are used in, while “sub-use” and “application” provide information as detailed as possible on the specific products or processes the substances are used in.

#### 2.1.3 Definition of Functions

For each application of PFAS, the chemical function, end-use function, and function as a service were determined, as defined in the functional substitution approach.^25^ In short, the chemical function is typically determined by the physicochemical properties of a substance, and it refers to the actual technical function provided by the substance to a product or a process, as defined in the OECD guidance on harmonized functional, product, and article use categories.^30^ The end-use function describes the specific properties the substance brings to the product or process due to its chemical functions. Function as a service describes the overall benefit that the substance in a specific product or process offers to society.^25^

#### 2.1.4 Definition of Alternatives

In this study, “alternative” refers to any other means to provide functions comparable to those of PFAS, by considering the chemical function, end-use function, and function as a service. The alternative can be another chemical substance (“alternative substance”) or material (“alternative material”), but it can also be a change in the formulation or design of the product (“alternative product”) or in the industrial process (“alternative process”) so that the chemical functions of PFAS are not required at all. Additionally, an alternative can be an entirely different technology (“alternative technology”) that provides similar services to the product/process with PFAS.

### 2.2 Overview of the Database

As illustrated in Figure 1, the database is structured around the uses of PFAS defined by the use category, sub-use, and application.

The PFAS used are identified with a substance name, a CAS number (Chemical Abstracts Service Number), and an elemental composition if it is available. Polymeric PFAS were automatically identified based on the occurrence of the word *polymer* in PubChem’s synonyms for the substance or the term *poly* in the substance IUPAC’s name. Polymers were then manually curated based on recorded structure, available name, and comparison with the dataset from Glüge et al.^2^

As previously mentioned, the chemical functions provided by PFAS were determined for each application based on the OECD guidance.^30^ The end-use functions and function as a service were determined by careful and pragmatic examination of the needs and tasks a chemical, product, or technology is intended to fulfill in each use case. If relevant, a qualitative description of the technical requirements that potential alternatives must meet is provided.

All alternatives listed in the database are identified by a name (e.g., substance name, product trade name), or by a general description of what the alternative is, if a detailed name is not given. Alternative substances were identified by using their IUPAC names. For alternative substances and materials, a CAS number is provided, if available. If available, the composition of alternative products with the names of the ingredients and their CAS numbers is provided in the Supporting Information (SI). Alternatives were classified according to four different categories depending on the general chemistry they are based on: general synthetic organic compound; organosilicon compound; natural-based compound and derivatives; and inorganic compound. “General synthetic compound” and “organosilicon compound” refer to substances that are man-made, while “natural-based compound and derivatives” refers to substances that are based on naturally occurring substances (e.g., cellulose). The classification was made based on the names of the alternatives. Similarly, nonpolymeric and polymeric alternatives were differentiated.

For the alternatives and the products’ ingredients identified by a CAS number, information regarding their classification under the Classification, Labeling, and Packaging (CLP) Regulation no. 1217/2008, and whether the substance has been identified as persistent, bioaccumulative, and toxic (PBT) is provided. A check was also conducted to determine if these substances are already listed in restrictive lists by using the SubsPort Plus database.^31^ Further information on the database is provided in the SI.

For each alternative, a qualitative assessment of potential performance loss compared with PFAS in the specific application is provided. Additionally, general information about the market availability of each alternative for a specific application is provided.

Table 1 presents a summary of the available information for each use of PFAS including the PFAS used, their functions, and potential alternatives.

**Table 1:** Type of Information Available in the Database for Each Application of PFAS

**List of PFAS used**	**Functions provided by PFAS**	**Potential alternatives to PFAS**
Substance name	Chemical function	Name of the alternative
CAS number (if available)	End-use function	CAS number (if relevant and available)
Elemental composition (if available)	Function as a service	Type of alternative
If the substance is a polymer	Performance requirements for alternatives (if relevant)	General chemistry description of alternative
Source of information	Source of information	If the alternative has been assessed for PBT (if relevant)
		If the alternative has been classified under CLP (if relevant)
		If the alternative is listed in the Substitution Support Portal (if relevant)
		Description of potential loss in performance
		Description of market availability
		Source of information

### 2.3 Literature Search Strategy

#### 2.3.1 Information on Uses of PFAS and Substance Identities

The use categories, sub-uses, and applications were determined based on the information in the general REACH restriction proposal as it provides a good overview of the different uses of PFAS based on an extensive literature search and interviews with various stakeholders. The list of uses for the restriction was slightly modified to add more information from a previous study^2^ and from the PFAS guide developed by ChemSec.^27^ The correspondence between the uses from the present study and from previous work is presented in the SI.

The list of PFAS used was mainly compiled using the information from Annex A of the general REACH restriction proposal.^32^ The list of substances was complemented with the information from Glüge et al.,^2^ following the correspondence presented in the SI.

#### 2.3.2 Search for Functions of PFAS

Generally, the functions provided by PFAS in each application were determined based on the information available in Annexes A and E of the general REACH restriction proposal.^32,33^ This was then complemented with the information contained in the OECD reports on specific uses of PFAS,^34,35^ Glüge et al.,^2^ and the ChemSec PFAS guide,^27^ when relevant.

For the specific case of the use of PFAS in firefighting foams, the REACH restriction on PFAS used in firefighting foams was analyzed to determine the functions provided by PFAS.^36^ For the use of PFAS in cosmetic products, their functions were determined by using the CosIng database.^37^ For PFAS used as active ingredients in pharmaceuticals (API), biocide products (BP), and plant protection products (PPP), their functions were determined by using the anatomical therapeutic chemical classification and defined daily dose (ATC/DDD) system from the World Health Organization (WHO) Collaborating Centre for Drug Statistics Methodology,^38^ the database of the European Chemicals Agency (ECHA) on information on biocide products,^39,40^ and the ECHA database on approved active substances for plant protection products,^41^ respectively.

#### 2.3.3 Identification and Evaluation of Alternatives

##### 2.3.3.1 Identification of Potential Alternatives

The initial list of alternatives to PFAS was built based on the information in Annex E of the general PFAS restriction proposal^33^ and on the restriction on PFAS used in firefighting foams.^36^ This list has been complemented with additional alternatives listed in the OECD reports on specific use cases of PFAS^34,35^ and in the ChemSec’s webtool Marketplace.^42^

For the specific case of PFAS used as an ingredient in cosmetic products, it has been considered that any substance listed in the CosIng database with the same function as PFAS could be considered as a potential alternative. However, due to the very high number of substances (more than 10,000 entries), those alternatives are not listed in the present form of the database. Similarly, any substance with a similar function as PFAS in the biocide and plant protection products databases were considered as potential alternatives to PFAS used in BP and PPP, respectively. A similar approach was taken to identify alternatives to PFAS used as an API: any substance with the same code as PFAS in the WHO ATC/DDD index was considered to be a potential alternative. Those potential alternative substances are also excluded from the database in its current form.

##### 2.3.3.2 Identification of the Composition of Alternative Products

To determine the composition of the alternative products, a Google search was performed by using the product trade name and the terms *sds* or *safety data sheet* to obtain the product safety data sheet. If it was not available, the type of chemistry used in the product was determined based on its description available on the website of the company selling the product.

##### 2.3.3.3 Hazard Characterization of Alternatives

Information on the classification of alternative substances under the European CLP Regulation, and potential evaluation of the substances to be considered as PBT under the REACH Regulation was collected from Annex E of the general PFAS restriction proposal.^33^ If the alternative is not mentioned in the annex, but identified by a CAS number, a search on the ECHA database was performed to determine whether the alternative has been classified under CLP and whether a PBT assessment has already been performed.^43^ A similar approach was followed for all of the components of the alternative products, which are identified by a CAS number.

Additionally, a search was performed in the SubsPort Plus database^31^ for all alternatives identified by a CAS number to determine whether some stakeholders (e.g., governmental authorities; companies; nongovernmental organizations) already identified potential concerns for those substances. Background information about the SubsPort Plus database is provided in the SI. A similar approach was followed for all the components of the alternative products which are identified by a CAS number.

##### 2.3.3.4 Evaluation for Potential Performance Loss

The change of performance of the alternatives compared to PFAS was evaluated based on the qualitative description provided in Annex E of the general PFAS restriction proposal^33^ and on the restriction on PFAS used in firefighting foams.^36^ For alternatives not listed in the general description, the change of performance was evaluated based on the alternative description available on the provider’s website. No in-depth literature search was performed at this stage. The feasibility of adopting a potential alternative in a product or process was not assessed, as it needs to be performed case by case and would exceed the scope of this study.

##### 2.3.3.5 Evaluation of the Market Availability

The market availability of the alternatives was also evaluated based on the qualitative description provided in Annex E of the general PFAS restriction proposal^33^ and on the restriction on PFAS used in firefighting foams.^36^ For alternatives not listed in the general restriction, the authors assumed that they could be considered as available on the market if they have a trade name and are listed on a private company website; otherwise, they were marked as “unclear”.

### 2.4 Analysis of the Data

#### 2.4.1 Overview of the Data Included in the Database

The data were analyzed using the Pivot Table tool of Microsoft Excel to provide an overall picture of the number of applications of PFAS, the number and type of PFAS used, the number of functions they provide, and the number and type of potential alternatives identified.

#### 2.4.2 Analysis of Alternatives to PFAS

##### 2.4.2.1 Safety Considerations

Alternatives identified by a CAS number were categorized for safety considerations based on the results from the search in the Substitution Support Portal. Any alternatives already listed in the Stockholm Convention,^17^ REACH Candidate List,^44^ REACH Authorisation List,^45^ list of restricted substances under REACH,^46^ and/or European Directive 2004/37/EC on carcinogens, mutagens, and reprotoxic substances at work^47^ were considered as a **regrettable substitute**, as those are substances already banned or about to be banned from the market. Any alternatives that are listed in one (or several) lists included in the Substitution Support Portal, other than the ones mentioned above, were considered as a **potential regrettable substitute**, as those substances are already raising concerns among stakeholders. Alternatives not listed in the SubsPort Plus database were considered as **substitutes without presently identified concerns**, as no concerns related to their uses were raised as of May 2023. For alternatives that are only listed in the EU cosmetic products prohibited substances list, or the EU BPR nonapproved list, it was necessary to check manually the specific application of PFAS they could be used in as it was considered that **in the vast majority of cases** cosmetic and biocide products are used with very specific exposure routes which may not be relevant for the specific use being considered. If the potential exposure routes were thought to be significantly different than those in cosmetic and biocide products, the alternatives were then considered as substitutes without presently identified concerns. A similar approach (i.e., to that used for classifying alternative chemicals) was taken to classify ingredients of alternative products identified by a CAS number.

##### 2.4.2.2 Performance Loss Considerations

Alternatives were categorized for the technical feasibility of substitution based on the information on potential changes in performance compared to PFAS. Four categories were created: (1) Category I gathers alternatives that provide similar or greater performance than PFAS; (2) Category II gathers alternatives that provide satisfactory performance for a limited range of environmental conditions (e.g., only for a certain temperature range) and the alternatives that provide one of the chemical functions of interests (for the cases where PFAS deliver several functions); (3) Category III gathers alternatives that do not provide satisfactory performance; (4) Category IV gathers alternatives for which more tests are still needed (as of March 2023) to conclude on their suitability to replace PFAS.

##### 2.4.2.3 Market Availability

A similar approach was taken to categorize alternatives based on the information on their availability on the market: (1) Category I gathers alternatives that are already available on the market and in use (as of March 2023); (2) Category II gathers alternatives that are available on the market and in use but only in a limited number of applications; (3) Category III gathers alternatives that are available on the market but not in use for the specific application which is being evaluated; (4) Category IV gathers alternatives that are not placed on the market yet (as of March 2023).

##### 2.4.2.4 Evaluation of Substitution Potential

**Figure 2:**
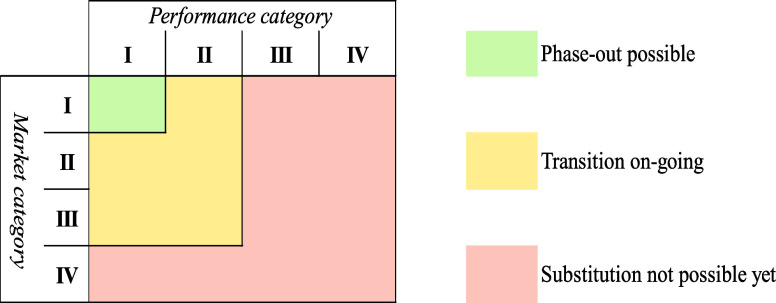
Substitution potential according to changes in performance and market availability of the alternatives.

The substitution potential was evaluated for each application based on the information related to the suitability of alternative and their availability on the market as described above and following the matrix in Figure 2.

### 2.5 Illustrative Case Study

The use of PFAS in fluorinated gases will serve as a case study to illustrate how the information contained in the database can be used. The detailed description of the case study can be found in the SI. The main findings will be briefly summarized in the Results and Discussion section.

## 3 Results and Discussion

The database has been freely available as an open data set on Zenodo since September 2023, and contingent on available funding it will be continuously updated as more information becomes available. The analyses presented here are based on the data accessible in the database as of April 20, 2024.

### 3.1 Number of Substances and Uses of PFAS Included in the Database

A total of 1697 individual substances defined as PFAS were identified in the database, out of which 1453 have a CAS number. 287 of the substances are classified as polymeric PFAS. Those numbers correspond well with the results of a similar study on the uses of PFAS which took a different approach.^2^ However, this number is much lower than the 10,000 PFAS, which is often quoted in the restriction reports,^48,49^ or the 6 800 PFAS listed in the ECHA database on CLP classifications. Furthermore, by using PubChem’s application programming interface^50^ to match CAS numbers of registered substances under REACH^51^ to structures that were identified as PFAS by a SMILES arbitrary target specification (SMARTS) match using RDKit,^52^ it was found that only 439 discrete fluorinated substances are registered under REACH (as of July 2023). This indicates that the majority of the thousands of PFAS mentioned in the restriction dossier are either single substances used in amounts below 1 ton per year in Europe, are a constituent of unknown or variable composition, complex reaction products, or biological materials (UVCB), which makes it difficult to properly identify them for registration, or are not covered by the Registration under the REACH Regulation (e.g., polymeric PFAS).

Table 2 presents an overview of the number of uses of PFAS included in the database as well as the number of functions provided by PFAS. Overall, the database contains 18 different use categories of PFAS, which are subdivided into 85 sub-uses and 325 applications. This number is higher than the approximately 200 uses listed in the study from Glüge et al.^2^ This difference is due to the way the database was built, as some applications of PFAS were duplicated, as they are common to several use categories. For instance, PFAS used for wires and cables insulation are listed in all use categories where cables are used (i.e., electronics and semiconductors sector; medical products; transport sector), which results in three distinct applications in this database, instead of only 1 from Glüge et al.^2^ The full list of applications of PFAS is provided in the SI.

**Table 2:** Overview Numbers on Uses of PFAS Included in the Database and the Functions They Provide

**Use categories**	**Sub-uses**	**Applications**	**Chemical functions**	**End-use functions**	**Services**
Active pharmaceutical ingredients (API)	2	14	1	24	21
Biocides (BP)	1	4	1	4	4
Building and construction products (Build.)	9	17	14	18	30
Consumer mixtures (Consu.)	7	15	7	15	12
Cosmetic products (Cosm.)	5	32	9	12	6
Electronics and semiconductors sector (Elec.)	3	29	17	22	37
Energy sector (Energy)	9	19	17	19	24
Firefighting foams (FFF)	1	5	1	1	3
Fluorinated gases (F-gases)	7	29	8	14	27
Food contact materials (FCM)	2	4	4	4	9
Industrial production (Indust.)	8	28	12	18	11
Lubricants (Lubr.)	3	42	11	13	19
Medical products (Med.)	6	21	14	18	29
Metal plating and metal products manufacture (Metal)	2	4	8	10	14
Petroleum and mining (Mining)	2	9	10	13	15
Plant protection products (PPP)	1	6	3	7	5
Textile, upholstery, leather, apparel, and carpets (TULAC)	7	20	11	15	21
Transport sector (Transp.)	10	27	16	24	45
**Total**	**85**	**325**	**39**	**131**	**201**

For 88 of those applications, the majority of the PFAS listed in the database are polymeric PFAS. It is important to note that given the structure of the database, it is not possible to determine the precise tonnages of PFAS used for each application. At best, one could refer to the PFAS restriction proposal under REACH to get an estimate of the tonnage band of PFAS used per use category listed in the database.^32^ According to the dossier submitters, fluorinated gases, the transport sector, and the textile, upholstery, leather, apparel, and carpets (TULAC) products are the use categories with the highest tonnages of PFAS used (i.e., with 543 568; 116 968 to 251 194; and 41 183 to 142 692 t/year used, respectively, in the EU alone). The estimations of volumes of PFAS used for each use category of the PFAS restriction proposal are available in the SI.

#### 3.1.1 PFAS Used in Fluorinated Gases

In total, 118 PFAS used in fluorinated gases are listed in the database, including three polymeric PFAS. The use category was subdivided into 7 subuses and 30 distinct applications. The full list of the subuses, applications, and PFAS used in fluorinated gases is provided in the SI.

### 3.2 Functions of PFAS

PFAS provide 39 different chemical functions, 131 different end-use functions, and 201 different functions as a service when considered in the context of specific applications. The most common chemical functions of PFAS are heat stabilizer, corrosion inhibitor, and waterproofing agent in 96, 91, and 75 applications, respectively. The main purpose of these functions in a specific application is to enhance the durability of the product or coating by providing resistance to water, extreme temperatures, and aggressive chemicals. It is important to note that PFAS with the same chemical function can provide a wide range of different services, within and across different use categories. For instance, PFAS are used as waterproofing agents in the coating of solar panels and wind turbines to protect them against the weather, but they can also be used in the internal sections of tubes and catheters to ensure the proper delivery of the liquid during medical treatments and prevent the proliferation of bacteria in the tubes. PFAS are also used as waterproofing agents in TULAC in consumer sportswear to protect the products against the weather, but also in home textiles to protect the products against humidity thus preventing the formation of mold. By considering the three different levels of functions of a substance of concern when defining its uses, it is then possible to identify all types of potential alternatives and go beyond only considering alternative substances with similar chemical functions.

Additionally, PFAS provides more than one chemical function, end-use function, and service in 161, 178, and 193 of the applications listed in the database, respectively. An overview of the number of functions delivered by PFAS per application is presented in the SI. For one single application, PFAS were shown to deliver up to eight chemical functions, eight end-use functions, and nine services. For instance, PFAS are used in the coating of solar panels as a binder and wetting agent to ensure a proper adhesion of the coating to the substrate and ensure that the coating is properly leveled and free of defects (e.g., cracking). They are also used as preservatives to prevent bacterial development on the surface of the panel and as a UV stabilizer, heat stabilizer, and waterproofing agent to provide resistance to light, high temperatures, and water to improve the durability of the coating and the panel against the weather conditions. At last, they are used as anti-adhesive agents to provide dirt repellence to the coating to make sure that the surface stays clean to improve the productivity of the panel. This emphasizes the challenges faced in substituting PFAS with only one alternative. It seems unlikely that one alternative can provide such a diversity of functions alone.

The information included in the database should allow the user to identify the functions delivered by PFAS which are critical for the technical performance of the application and differentiate them from the functions which are “nice to have”. As highlighted by previous studies, identifying the uses of a substance of concern that are “fit-for-purpose” in the considered application is a critical step when trying to identify potential alternatives.^26,53,54^ Such an assessment was not undertaken in this study and should be the focus of further work, for instance, by getting more specific details on one or two specific uses of PFAS.

#### 3.2.1 PFAS Used in Fluorinated Gases

Overall, fluorinated gases provide 8 different chemical functions, 14 end-use functions, and 27 services across the 30 applications in which they are used. As an example, fluorinated gases are used in refrigeration and heat pumps as a heat transferring agent for providing or removing heat from a space (depending on the specific application) to increase or decrease the temperature in a building or transportation mode (in the case of heat pumps and air conditioning, respectively) but also to ensure the preservation of goods in domestic, commercial, and industrial refrigeration (e.g., fridges, cool rooms). They are also used as a heat transferring agent for the cooling of electronics (e.g., in data centers or car batteries). Some fluorinated gases are used as a *foamant* to ensure the expansion of foams which are used as insulation material to ensure thermal insulation of e.g. buildings. They are also used in rigid polyurethane pipe-in-pipe foams to prevent pipes from freezing and cracking. The full list of functions provided by fluorinated gases for each application is provided in the SI.

### 3.3 Alternatives to PFAS

For the specific cases where PFAS are used for their biological activity (referred to as “active substance”), and as ingredients in cosmetic products, data related to their potential alternatives is not included in the following analyses. Active substances in biocides, pharmaceuticals, and plant protection products are used for their specific biological mode of action. Although other substances have been identified that have similar general adverse effects as PFAS in the different databases,^38,39,41^ those “effect categories” were too broad to determine whether all active substances listed could be used as substitutes to provide the same expected outcome which makes it difficult to determine if they would be suitable alternatives. Regarding ingredients in cosmetic products, a previous study suggested that any substance listed in the CosIng database with a similar function as the substance to substitute could be considered as a suitable alternative.^54^ Given that the CosIng database is publicly available and given the very high number of substances that it would represent (in order of several thousands), those alternative ingredients were not added to the database.

In total, 530 different alternatives have been identified and are listed in the database for the 14 other use categories before any evaluation for their suitability. An overview of the number and the type of alternatives to PFAS that have been identified for each use category is presented in the SI. The database lists 162 alternative substances, 163 alternative materials, 128 alternative products, 37 alternative processes, and 40 alternative technologies. Based on the information collected, PFAS can be phased out in four applications, which do not require the service they provide, namely, in coating for strings of musical instruments,^33^ in certain food packaging when uncoated paper and uncoated paper plates are suitable alternatives,^55,56^ and in lubricants for certain consumer products (e.g., bike chain lubricant).^57^ No alternatives have been identified for 83 applications, including 25 applications in industrial uses, for example, in the chemical industry (e.g., as solvent; for polymer curing), or for the production of plastic and rubber (e.g., as processing aid; as mold release agent). The full list of applications with no alternatives identified is provided in the SI.

It is important to note that the list of alternatives to PFAS that have been used to build the database is not exhaustive. It has been created largely based on the information available in the REACH PFAS restriction proposal, which summarizes the main findings of the dossier submitters following a review of the available literature and interviews with the relevant stakeholders. It does not include new information received by the dossier submitters during public consultation. Furthermore, no interviews with alternative producers or users were performed as it goes beyond the scope of this study. Further work is needed to analyze such comments to determine whether new potential suitable alternatives to PFAS are available based on the relevant information, which was submitted. Additionally, the database presents a general description of the alternatives but does not provide information on the potential providers of those alternatives. The ChemSec webtool Marketplace^42^ is currently the best online tool for finding information on the providers of alternatives.

The main goal of this study is to present an overview of the types of alternatives to PFAS and their availability. No in-depth literature review has been performed to attempt to identify additional potential alternatives so far. It would be a very time-intensive task (e.g., literature and patent search) to investigate all potential alternatives for all uses of PFAS. This should be the focus of further detailed work and will likely be largely undertaken by companies that aim to replace PFAS in specific uses.

#### 3.3.1 PFAS Used in Fluorinated Gases

60 alternatives to fluorinated gases are listed in the database. These are mainly alternative substances with similar chemical functions (e.g., hydrocarbons as heat-transferring agents). However, the database also lists 12 alternative materials, 8 alternative processes, and 7 alternative technologies that do not require the use of fluorinated gases. For instance, PFAS used as foam blowing agents in thermal insulation foams could be replaced by other blowing agents (e.g., CO_2_ or pentane), or the whole foam could be replaced by another insulation material that does not require the use of blowing agents (e.g., fiberglass or Rockwool). This emphasizes the need to properly identify the functions of a substance of concern and its true purpose in a specific application to identify all potential alternatives. The whole list of alternatives to fluorinated gases for each application is provided in the SI.

### 3.4 Assessment of Alternatives to PFAS

#### 3.4.1 Safety Considerations

In total, 186 alternatives were screened for potential concerns, of which 10 are already restricted, or about to be restricted, and were considered to be regrettable substitutes. 58 of the evaluated alternatives were identified as of potential concern by some stakeholders, and further assessment should be performed to determine whether they are truly raising concerns for the environment and for human health, and further actions should be taken to phase out the alternatives that represent a risk as soon as possible. 130 alternatives are not listed in the SubsPort Plus database and were identified as substitutes without presently identified concerns. An overview of the potential for each alternative to PFAS to be a regrettable substitute within each use category is presented in the SI.

The approach taken in this study to screen for potential hazards related to the use of the potential alternatives aims to provide an overview of what is known about potential concerns associated with the identified alternative substances. It does not provide a full hazard characterization of the identified alternatives. In other words, alternatives identified as “substitutes without presently identified concerns” are not necessarily safe. The approach taken only determines that no concerns related to their uses have been raised in the SubsPort Plus database yet. In addition, it was possible to do the assessment only for the alternatives identified by a CAS number, which represents 35% of the total number of alternatives listed in the database. Regarding the alternative products, it was possible to determine the composition for only 21 products out of a total of 116, which are included in the database based on the available safety data sheets. Furthermore, no information related to other environmental impacts (e.g., global warming potential; fossil resources used) is provided in the database. It is very common in substitution practices that very little is known about the potential hazards and risks of an alternative.^58^ This data gap emphasizes the need for further work to properly characterize the hazard profile of the identified alternatives to ensure that regrettable substitution can be avoided.

Only 31% of the identified alternatives are other chemical substances. As already highlighted in previous studies, new alternative assessment methods are needed to evaluate and compare different types of alternatives.^59^ So far, alternative assessment methods, which have been developed to evaluate the hazards of alternatives, are well suited for comparing different chemical substances but not for comparing different materials or technologies. The development of novel methods based on life-cycle thinking and considering a wide range of different environmental impacts should be the focus of further work in order to prevent or minimize “problem shifting”, i.e., reducing chemical hazards but simultaneously increasing impacts on climate.

#### 3.4.2 Substitution Potential of PFAS

**Figure 3:**
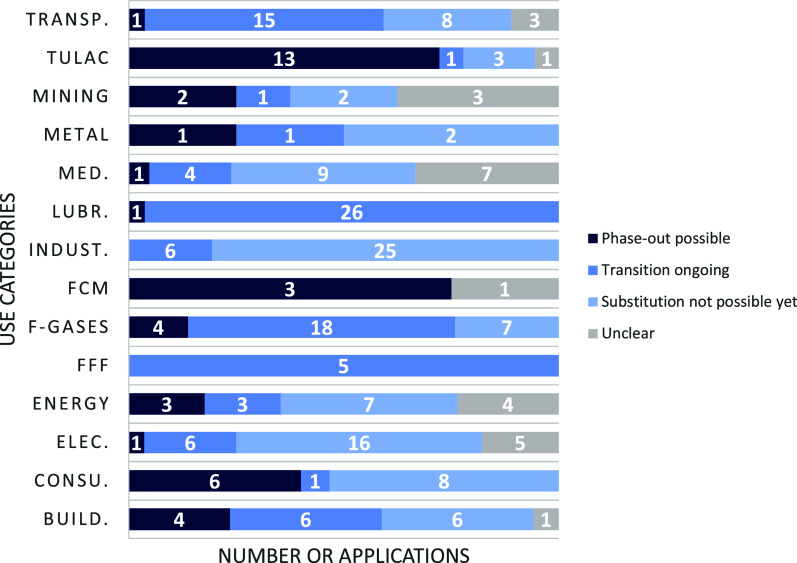
Substitution Potential of PFAS.

Figure 3 shows the potential for substituting PFAS in each use category. A comparison of the performance of the identified alternatives with that of PFAS and their availability on the market has led to the conclusion that technically feasible alternatives to PFAS are available for 40 applications as of April 2024. For 93 applications, potential alternatives have been identified at the time of the analysis, but more time and more information are required to ascertain the suitability of these alternatives, either because further tests are necessary to ensure their efficacy or because they have not yet become sufficiently established on the market (or both). At last, for 93 applications, either no suitable alternatives have been identified at the time of the analysis or the alternatives that were identified are still under development, and they are not available on the market yet. As no information on the amount of PFAS used for each application is available, it is unfortunately not possible to evaluate the total tonnage, which can already be phased out at this stage. This is an area that requires further investigation.

Alternatives were classified in the first category only if they are capable of providing the same level of technical performance as PFAS. However, in some cases, such a high level of performance is not needed for the final product to fulfill its intended service. In such instances, rather than attempting to match the same level of performance as PFAS, the assessor should develop a range of performance criteria to differentiate between the alternatives that are “sufficiently performant”, from the alternatives that are “best in class”. For example, all identified alternatives to PFAS in firefighting foams were classified in category 2 because they do not meet the strict technical requirements for some sectors (e.g., for military applications), despite their ability to extinguish a fire. A previous study on firefighting foams used for military applications in the US demonstrated that the strict regulatory requirements were largely based on foams containing PFAS with minimal flexibility which makes it very difficult for PFAS-free to be considered as suitable.^53,60^ However, the authors argued that the alternative foams are “sufficiently performant” to extinguish fires and that the regulatory requirements should be revised to support the use of PFAS-free alternatives.^53^ It is recommended that the performance of the alternatives in the database should be evaluated in a similar way on a case-by-case basis before a final conclusion can be reached regarding their suitability. The information available in the database can be used as a first step to identify potential substitutes as part of an alternative assessment. It is unlikely that the alternatives listed in the database will be simultaneously safer for human health and the environment, cheaper, and just as performant as PFAS. Proper alternative assessments for specific uses should still be performed to evaluate the potential trade-offs of phasing out PFAS.

At this point, the main goal of the database is to provide an open platform on which information on the availability of PFAS-free alternatives is freely available. Such efforts could be useful for companies willing to phase out their uses of PFAS by providing them with information on the types of alternatives that are already in use. Furthermore, the information in the database can help the authorities to identify uses of PFAS where alternatives are still lacking and should be the focus of their time and resources for further research. Although the database has been built based on information from a European perspective, the platform can still be useful for authorities from other regions of the world to reduce duplication of efforts to transition toward PFAS-free alternatives.

### 3.5 Potential Applications and Limitations of the Database

The database presented in this study aims to present an overview of what is known about the uses of PFAS, their functions, and the availability of potential alternatives. The list of identified alternatives in the database is not exhaustive, and further work is needed to perform deeper searches in the literature to find other potential suitable alternatives. Similarly, the information on the safety and suitability of the alternatives available in the database is very succinct at this stage. We believe that the information available in the database can help a company willing to substitute their use of PFAS in the initial phase of their substitution activities, but a proper assessment of alternatives is still needed to ensure that the alternatives are suitable for their specific use.

The information in the database can also be useful to identify uses of PFAS that are not essential for society. The essential-use concept was first introduced in the Montreal Protocol in 1987 to guide the phasing-out of ozone layer-depleting substances.^61^ The Parties agreed that a “controlled substance should qualify as “essential” only if (1) it is necessary for the health, safety or is critical for the functioning of society (encompassing cultural and intellectual aspects); and (2) there are no available technically and economically feasible alternatives or substitutes that are acceptable from the standpoint of environment and health”.^62^ In April 2024, the European Commission published a guidance on criteria and principles for the essential-use concept in the EU chemical regulations to guide the phasing-out of the most harmful chemicals.^63^ In summary, three main questions should be answered in an essentiality assessment: (1) Is the chemical function of the most harmful substance needed for the final product to deliver its service? (2) Does the use of the most harmful substance fulfill at least one of the criteria listed in the guidance to be considered as necessary for health and safety or critical for the functioning of society? (3) Are safer alternatives capable of providing similar functions and a sufficient level of performance available?^63^

As explained previously, the information provided in the database can help identify applications of PFAS for which at least one suitable alternative is already available on the market. By combining this information with the safety assessment of the potential alternatives to ensure that they do not present a risk for regrettable substitution, it is possible to identify the non-essential uses of PFAS, as of April 2024.

Out of the 40 applications for which the phase-out of PFAS is possible, alternatives for which no concerns have been presently identified are available for 28 applications. Therefore, approximately 10% of the uses of PFAS included in the database (*n* = 325) can be considered non-essential because safer suitable alternatives are available and should not be derogated from regulations. For 4 applications, the only suitable alternatives that have been identified are already raising concerns among various stakeholders. For instance, 4 suitable and available alternatives to PFAS used as fluorinated gases for the immersion cooling of electronics have been identified, namely, ammonia (CAS: 7664–41–7), isobutane (CAS: 75–28–5), *n*-butane (CAS: 106–97–8), and propane (CAS: 74–98–6). However, concerns have been raised by governmental agencies (e.g., Canadian Environmental Protection Agency; US Environmental Protection Agency; Swedish Chemical Agency; German Environment Agency), nongovernmental organizations (e.g., European Trade Union Institute), and companies (e.g., Bluesign Technologies) regarding the use of all identified alternatives. A proper hazard assessment of those alternatives should be performed to ensure that they are safer than PFAS before concluding the essentiality of the use of PFAS in order to prevent regrettable substitution. For the eight remaining applications, the safety of the identified alternatives could not be evaluated in this study. The full list of the applications that have been evaluated for essentiality based on the availability of safer alternatives is presented in the SI.

As highlighted in previous studies, defining the use of a substance of concern following the functional substitution approach can be helpful in evaluating its essentiality.^26,54^ Therefore, the database can be used to identify applications where the chemical function provided by PFAS is not needed in the final product to deliver its services, and those that do not fulfill the criteria to be considered as necessary for health and safety, or critical for the functioning of society. For instance, the use of PFAS in bike chain lubricants is not essential as the function provided by PFAS is not necessary for the technical performance of the final product, as already demonstrated in a previous study.^57^ PFAS are also used as wetting agents in anti-fog sprays to minimize the condensation of water vapor and therefore prevent “fogging” on a surface (e.g., in swimming goggles). Although PFAS are necessary for the technical performance of the final product in that case, this particular service does not fulfill any of the criteria listed by the European Commission^63^ to be considered as necessary for health and safety or critical for the functioning of society. In both examples, the use of PFAS can be considered as non-essential and could be phased out even though no suitable alternatives have been identified at the time of the study. Such a reasoning could be followed for other uses included in the database for which no suitable alternatives have been identified to prevent wasting time and resources on trying to find alternatives for uses of PFAS that are not the most critical for society.

## Data Availability

The database of alternatives to PFAS that has been built in this article is freely available as a data set on the Zenodo platform via the following link: https://zenodo.org/doi/10.5281/zenodo.8434809.

## Supporting Information Available

Additional details on the methods followed to build up the database and additional analysis of the data on alternatives to PFAS, including extracts of the database (PDF)
Applications of PFAS listed in the database; links between applications of PFAS listed in the database and the uses identified by Glüge et al.; composition of alternative products to uses of PFAS; list of applications of PFAS without identified alternatives; list of PFAS used as fluorinated gases; list of functions delivered by PFAS used as fluorinated gases; and list of alternatives to PFAS used as fluorinated gases (XLSX)
