# Supplementary material for: An Overview of Potential Alternatives for the Multiple Uses of Per- and Polyfluoroalkyl Substances
Source: Environ Sci Technol. 2025 Jan 24;59(4):2031–42. doi: 10.1021/acs.est.4c09088 (PMC11800378; doi:10.1021/acs.est.4c09088)
Supplement: Supplementary file 1 — es4c09088_si_001.pdf [file es4c09088_si_001.pdf]

Supporting information to: An overview of potential alternatives for the multiple uses of per- and polyfluoroalkyl substances (PFAS)

Romain Figuière*[1], Luc T. Miaz[1], Eleni Savvidou[1], Ian T. Cousins[1].

* : Corresponding author – romain.figuiere@aces.su.se

1: Department of Environmental Science, Stockholm University, SE-10691 Stockholm, Sweden

Summary

Number of pages: 8

Number of Tables: 9 (7 are provided in the separate Excel files named *"Supporting data"*

Number of Figures: 3

Contents

SI 1: The Substitution Support Portal S1

SI 2: List of applications of PFAS S4

SI 3: Links with uses from Glüge et al. (2020)[2] S4

SI 4: Tonnages of PFAS used per uses included in the REACH Restriction dossier S5

SI 5: Composition of alternative products S6

SI 6: Number of functions delivered by PFAS S6

SI 7: Overview data on the identified alternatives S7

SI 8: Case study – PFAS used as fluorinated gases S8

References S8

Table of Figures

Figure SI-6 1 Number of functions delivered by PFAS per applications S6
Figure SI-7. 1 Number and types of identified alternatives to PFAS per use categories S7
Figure SI-7. 2 Potential for regrettable substitution S7

Table of Tables

Table SI-1 1 List of restricted substances lists in the Substitution Support Portal database S3
Table SI-4 1 Estimations of volumes of PFAS used according to the dossier submitters S5

Note: In order to save space in this document, the tables listed below are accessible in the separate Excel file named "Supporting data"

Table SI-2.1 Applications of PFAS listed in the database S4

Table SI-3.1 Links between applications of PFAS listed in the database and the uses identified by Glüge et al. (2020) S4

Table SI-5.1 Composition of alternative products to uses of PFAS S6
Table SI-7.1 List of applications of PFAS without identified alternatives ……………………………..S8
Table SI-8.1 List of PFAS used as fluorinated gases S8
Table SI-8.2: List of functions delivered by PFAS used as fluorinated gases S8
Table SI-8.3: List of alternatives to PFAS used as fluorinated gases S8

SI 1: The Substitution Support Portal (SubsPort Plus)

The Substitution Support Portal is the final result of the SUBSPORT project which was part of the LIFE+ Program of the European Union. Since 2013 the Portal has offered a wide range of information to guide substitution activities to phase-out the use of hazardous chemicals. The German Federal Institute for Occupational Safety and Health (BAUA) has taken the responsibility to develop the Portal further and to keep it up to date[1].

Among others, the Substitution Support Portal presents a database of hazardous substances whose uses are restricted be legal or voluntary obligations from various stakeholders (i.e. “List of lists”), freely accessible on the website via this link: https://www.subsportplus.eu/subsportplus/EN/Substances/Database-of-restricted-and-priority-substances/restricted-priority-substances_node. The relevant stakeholders could be different national or international (e.g. European) authorities, companies, or non-governmental organizations.

In total, the database contains the 38 lists of substances which are listed in Table SI-1.1 below[1].

Table SI-1 1 List of restricted substances lists in the Substitution Support Portal database

List name	Type of list	Link
Boots: Priority Substances List, Materials Restricted for Use	Company of Sector Lists	Link
Dell: Restricted Materials Guidance Document	Company of Sector Lists	Link
Volvo's Grey List: Chemical substances whose use within the Volvo Group shall be limited	Company of Sector Lists	Link
Volvo's Black List: Chemical substances which must not be used within the Volvo Group	Company of Sector Lists	Link
Global Automotive Declarable Substance List	Company of Sector Lists	Link
BSSL Bluesign System Substances List	Company of Sector Lists	Link
OEKO-TEX Standard 100	Company of Sector Lists	Link
Zero Discharge of Hazardous Chemicals Programme: Manufacturing restricted substances list	Company of Sector Lists	Link
Vestas Blacklist	Company of Sector Lists	Link
H&M Group: Chemical Restrictions	Company of Sector Lists	Link
SC Johnson Green List	Company of Sector Lists	Link
Nokia Substance List	Company of Sector Lists	Link
REACH Candidate List	EU Regulatory Lists	Link
REACH Authorisation List	EU Regulatory Lists	Link
REACH Restriction List	EU Regulatory Lists	Link
List of non-approved substances under Biocidal Products Regulation	EU Regulatory Lists	Link
List of prohibited substances in Annex II of Cosmetic Products Regulation	EU Regulatory Lists	Link
Water Framework Directive, List of Priority Substances	EU Regulatory Lists	Link
EU Regulation on Persistent Organic Pollutants	EU Regulatory Lists	Link
RoHS Directive	EU Regulatory Lists	Link
KEMI: PRIO Database - List of phase-out substances	Governmental Lists	Link
Canadian Environmental Protection Agency: Priority Substances List	Governmental Lists	Link
US Environmental Protection Agency: Extremely Hazardous Substance List	Governmental Lists	Link
Master List of German Federal Environment Agency	Governmental Lists	Link
Massachusetts Toxics Use Reduction Act (TURA) Chemicals List	Governmental Lists	Link
California Proposition 65 List	Governmental Lists	Link
Stockholm Convention on Persistent Organic Pollutants	International Agreements	Link
OSPAR List of chemicals of possible concern	International Agreements	Link
OSPAR List of chemicals for priority action	International Agreements	Link
Trade Union Priority List for REACH Authorisation	NGO or Trade Union Lists	Link
ChemSec SIN List	NGO or Trade Union Lists	Link

Substance Database according to SUBSPORTplus screening criteria	SUBSPORT	Link

The last update of this database was in May 2023.

SI 2: List of applications of PFAS

Table SI-2.1, which is accessible in the separate Excel file “Supporting data”, lists all the identified applications for each use category.

Table SI-2.1 Applications of PFAS listed in the database

SI 3: Links with uses from Glüge et al. (2020)[2]

To the best of our knowledge, Glüge et al. (2020) made the first attempt to map out all uses of PFAS. The approach taken in this study slightly differed from theirs and this requires explanation. In particular, the categorization and denomination of PFAS uses were different. In order to use the information from Glüge et al. (2020), uses from their database were manually matched with the applications identified in this study based on the use category, sub-use and applications names. If no matches were found, a new application was created by keeping the denomination of Glüge et al. (2020).

Table SI-3.1, which is accessible in the separate Excel file “Supporting data”, presents all links between the two databases.

Table SI-3.1 Links between applications of PFAS listed in the database and the uses identified by Glüge et al. (2020)

36 uses from Glüge et al. (2020) could not be assigned to any applications in this study. However, those uses were considered to be minimal in terms of number of PFAS and were therefore not included in the database.

SI 4: Tonnages of PFAS used per uses included in the REACH Restriction dossier[3,4]

The table SI-4.1 below presents the estimated volumes of PFAS used per use categories of the REACH Restriction, according to the dossier submitters.

Table SI-4 1 Estimations of volumes of PFAS used according to the dossier submitters

Use category	Tonnage band (tonnes/year)	Comments
**Active pharmaceutical ingredients**	500	
**Biocides**	Not available	No data on tonnage available
**Building and construction products**	5 241 – 12 725	
**Consumer mixtures**	2,8	Highly uncertain - 1,2 t/y used in the impact assessment and 1,6 t/y for uses in ski waxes
**Cosmetic products**	0,028 - 64,2	
**Electronics and semiconductors sector**	2 541 – 6 304	
**Energy sector**	2 884 – 3 214	
**Firefighting foams[4]**	480 – 560[4]	
**Fluorinated gases**	543 568	Sum of the tonnage in stocks, manufactured product and decommissioning, as detailed in table A.36
**Food contact materials**	18 597 – 29 772	
**Lubricants**	1 171 – 2 160	
**Manufacture of fluoropolymers**	Not available	
**Medical products**	24 672 – 61 527	
**Metal plating and metal products manufacture**	962 – 1 017	
**Petroleum and mining**	1 509,5 – 3 504,4	
**Plant Protection Products**	5 479	
**Textile, upholestry, leather, apparel, and carpets**	41 183 – 142 692	
**Transport sector**	116 968 – 251 194	Takes into consideration the PFAS used in cars already on the market, and the yearly usage of PFAS as detailed in table A.42

SI 5: Composition of alternative products

Table SI-5.1, which is accessible in the separate Excel file "Supporting data", presents the composition of the identified alternative products to uses of PFAS based on the information available in their safety data sheets. The table also presents the classification of the ingredients identified by a CAS number according to the CLP Regulation, if they have been evaluated as persistent, bioaccumulative and toxic (PBT), and if they are listed in the database of the Substitution Support Portal (the specific lists are specified if it is the case).

Table SI-5 Composition of alternative products to uses of PFAS

SI 6: Number of functions delivered by PFAS

Figure SI-6.1 illustrates the number of different functions which could be delivered by PFAS per application and, for each level of function considered (i.e. chemical function, end-use function, and function as a service). Furthermore, it states the number of applications included in the database with the same number of different functions. For instance, it can be read from the figure that PFAS deliver 2 different chemical functions, end-use functions, and services in 56, 52, and 69 applications, respectively. It can also be read that PFAS deliver up to 9 different services for two applications.

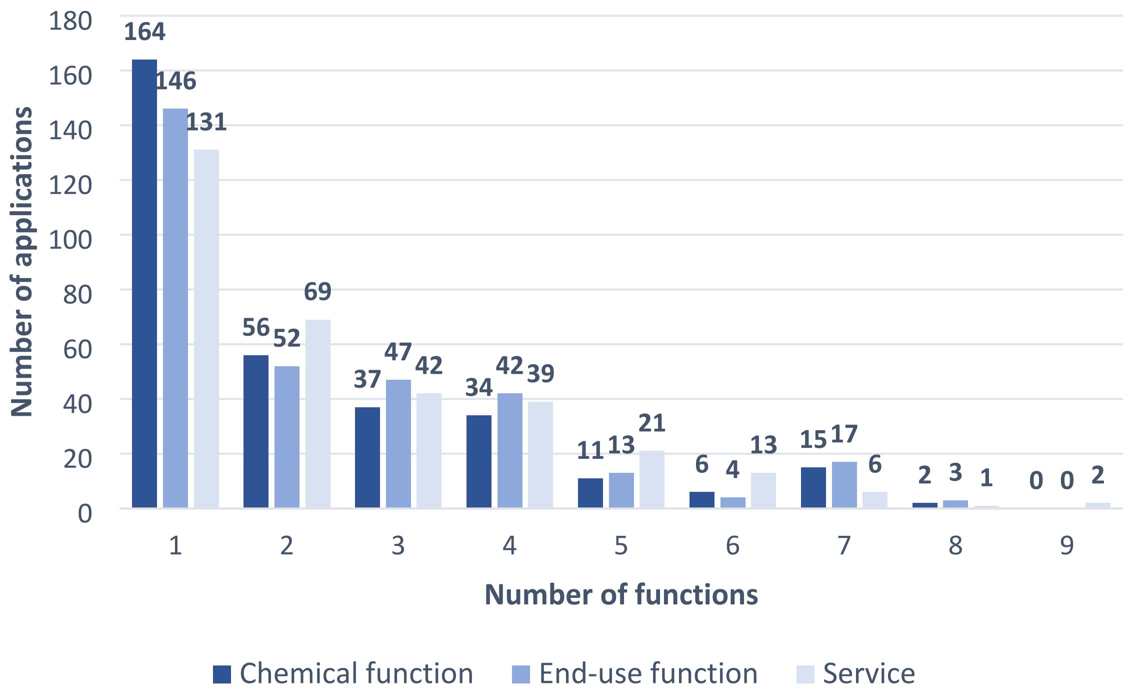

Figure SI-6 1 Number of functions delivered by PFAS per applications

SI 7: Overview data on the identified alternatives

Figure SI-7.1 presents the number and the type of the alternatives to PFAS which have been identified for each use category, while figure SI-7.2 illustrates the potential for each alternative to PFAS to be a regrettable substitute within each use category.

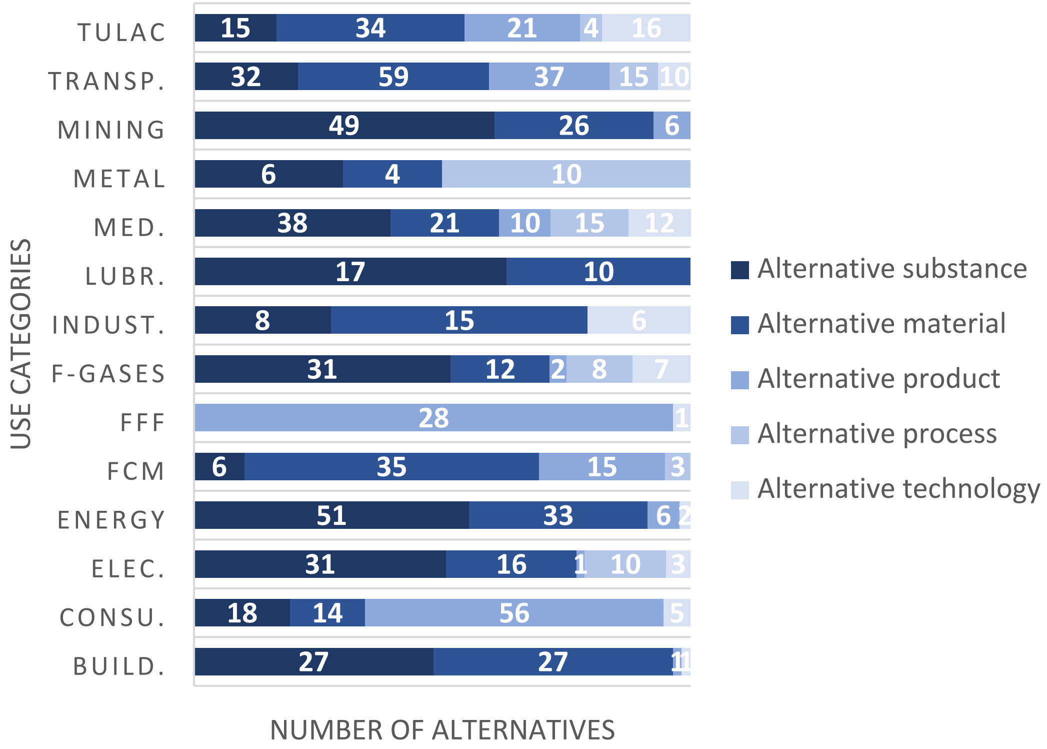

Figure SI-7. 1 Number and types of identified alternatives to PFAS per use categories

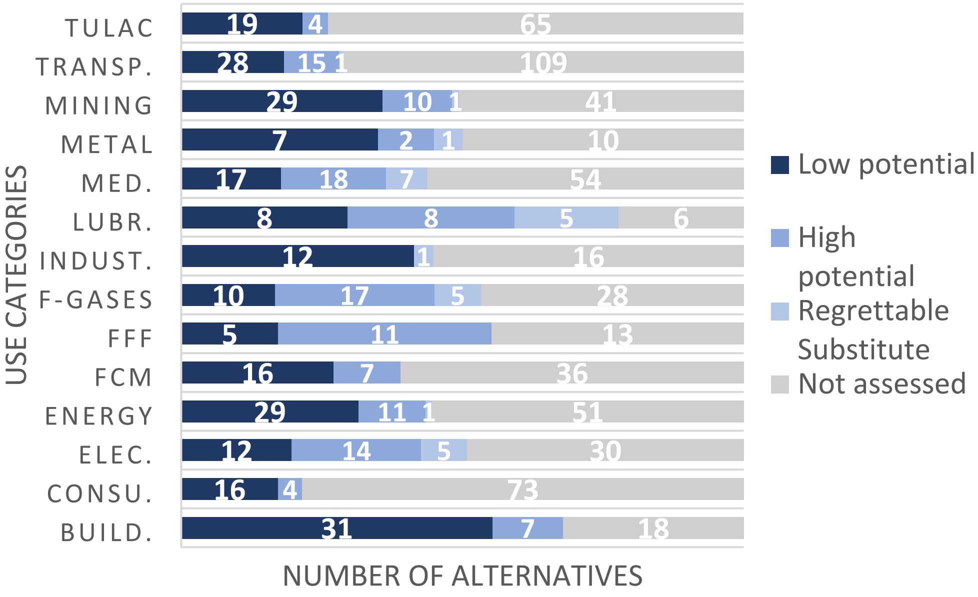

Figure SI-7. 2 Potential for regrettable substitution

Table SI-7.1, which is accessible in the separate Excel file "Supporting data", is an extract of the database listing the applications of PFAS for which no alternatives have been identified at the time of the study (as of April 2024).

Table SI-7.1 List of applications of PFAS without identified alternatives

SI 8: Case study – PFAS used as fluorinated gases

Tables SI 8.1, 8.2 and 8.3, which are accessible in the separate Excel file "Supporting data", are extracts from the database to illustrate how the information is presented. Table SI 8.1 lists the PFAS used as fluorinated gases, Table SI 8.2 presents the functions they provide in the different applications, and Table SI 8.3 lists potential alternatives for those applications.

Table SI-8.1 List of PFAS used as fluorinated gases

Table SI-8.2 List of functions delivered by PFAS used as fluorinated gases

Table SI-8.3 List of alternatives to PFAS used as fluorinated gases

References

(1) Bundesanstalt für Arbeitsschutz und Arbeitsmedizin (BAuA). *SUBSPORTplus - Database of restricted and priority substances -*. https://www.subsportplus.eu/subsportplus/EN/Substances/Database-of-restricted-and-priority-substances/restricted-priority-substances_node (accessed 2024-07-12).
(2) Glüge, J.; Scheringer, M.; Cousins, I. T.; DeWitt, J. C.; Goldenman, G.; Herzke, D.; Lohmann, R.; Ng, C. A.; Trier, X.; Wang, Z. An Overview of the Uses of Per- and Polyfluoroalkyl Substances (PFAS). *Environ. Sci. Process. Impacts* **2020**, *22* (12), 2345–2373. https://doi.org/10.1039/D0EM00291G.
(3) European Chemicals Agency. *Annex A to the Annex XV Restriction Report for the Restriction on the Manufacture, Placing on the Market and Use of PFASs*; ECHA: Helsinki, 2023. https://echa.europa.eu/registry-of-restriction-intentions/-/dislist/details/0b0236e18663449b (accessed 2023-07-10).
(4) European Chemicals Agency. *Annexes to the Annex XV Restriction Report on Proposal for Restion on Per- and Polyfluoroalkyl Substances in Firefighting Foams*; ECHA: Helsinki, 2022. https://echa.europa.eu/documents/10162/d66bdab3-fdb3-93ba-ee81-1dba3977f36d (accessed 2023-09-22).
